# Preventing childhood drowning in New York: a state action plan

**DOI:** 10.1186/s40621-026-00686-1

**Published:** 2026-05-19

**Authors:** Joyce C. Pressley, Marrick Anne McDonald

**Affiliations:** 1 Mailman School of Public Health, Department of Epidemiology, NY, USA; 2 Mailman School of Public Health, Department of Health Policy and Management, NY, USA; 3 The Center for Injury Science and Prevention, Columbia University in the City of New York, 722 168th Street, Room 510, NY, 10024 USA; 4 New York State Temporary Commission to Prevent Childhood Drowning, Newport, NY USA

**Keywords:** Childhood drowning, Drowning prevention, Drowning countermeasures, Storm flooding, Coastal surge, Standards, Swimming lessons, Water proficiency, water competency, Situational knowledge

## Abstract

The Temporary Commission to Prevent Childhood Drowning (NYS Drowning Commission) was signed into law by the New York State (NYS) Governor to address the public health issue of childhood drownings. This article documents the NYS Drowning Commission’s formation and highlights findings and recommendations contained in the 339-page final report. The comprehensive report found that both children and adults face evolving water-related risks associated with the state’s geography of extensive coastlines, rivers, lakes, and increasing exposure to extreme weather and flooding. Legislation that created the NYS Drowning Commission stipulated five major goals. In fulfillment of these goals, NYS Drowning Commission formulated more than 190 recommendations toward creation of a unified, multi-pronged, equity-centered culture of water safety and a NYS Office of Water Safety to provide coordination across the multiple agencies and sectors responsible for water safety. Short-term and long-term strategic solutions are recommended including building and upgrading physical pool infrastructures, adoption of advanced technologies, enhancement of the early warning system for dams and levees, public awareness campaigns that address water proficiency, water competence and situational knowledge of water, and aquatic training for physical education teachers. Other recommendations address standardized ages for initiation of water safety education, aquatic learning standards, vulnerable/disabled populations, data needs, community public-private partnerships, and gaps in services for underserved and/or under-resourced populations. Several water safety-related topics, including flood zones, storm surge areas, low-lying regions, aging infrastructure such as dams, hazardous coastal infrastructure, and climate-related hazards outside the scope of the NYS Drowning Commission are identified.

## Introduction

### Scope of the problem

In the U.S., drowning is the number one cause of death in children ages 1–4 years and the second leading cause of death in ages 5–14 years [1]. Although the all age unintentional drowning rate of New York State (NYS) residents is relatively low at 1.09 per 100.000 people, 761 New Yorkers drowned between 2000 and 2024, making it the fifth highest state for number of drowning deaths [2]. Compared to drowning in non-Hispanic white children, drowning risk is 2.9 times higher in non-Hispanic Hawaii Native and Pacific Islander children, 1.9 times higher in African American children and 1.5 times higher in American Indian/Alaskan Native children [2]. Additionally, special populations, such as those with autism, mental health and chronic conditions, have elevated risk of drowning [3, 4]. Drowning deaths are particularly high in children under the age of 15 with autism spectrum disorder who are reported to be 160 times more likely to die from drowning than other similarly-aged children [3]. 

### Environmental context

New Yorkers have multiple exposures to water throughout the state. The perimeter of NYS is surrounded by 2,625 miles of coastal waterways; NYS has coastal territory in the Atlantic Ocean and two Great Lakes: Lake Erie and Lake Ontario. NYS has more than 7,600 freshwater lakes, ponds and reservoirs, and more than 70,000 miles of rivers and streams [5, 6]. There are 51 Department of Environmental Conservation Parks, 181 State Parks, including Niagara Falls, and multiple municipal swimming pools and water parks. Childhood drownings have also occurred when children accessed bathtubs, standing rainwater, and decorative home landscape ponds. New Yorkers installed more backyard pools during the Covid-19 pandemic than at any time previously. [5, 6]

Several climate events have resulted in child and adult drownings in flooding basement apartments, on highways, and in coastal homes. [7, 8]. Communication during these events is challenging as approximately 30% of New Yorkers speak a language other than English at home and more than 2.5 million are reported to have limited English proficiency [5]. Climate events, such as Hurricane Ida in 2021, resulted in 13 direct deaths, 11 of which occurred from drowning in NYC basement apartments [7]. Unexpected flash floods in NYC in 2025 led to two more deaths in basements [8]. These deaths impacted immigrant and low-income households disproportionately. [7, 8]. Among households with incomes less than $50,000, 79% (79%) of children are reported to have few to no swimming skills [3–5, 7]. 

### Organizational context

The approach to water safety varies by regions across NYS. As of December 2025, three NYS aquatic governing agencies were engaged in the authorization and operation of aquatic facilities: (1) New York State Department of Health; (2) New York State Department of Environmental Conservation; and (3) New York State Office of Parks, Recreation, and Historic Preservation. The need to develop a cohesive, collaborative approach to water safety in NYS led to passage of legislation to address gaps and to advance and modernize NYS’s strategic approach to drowning prevention [5]. 

### Birth of the NYS drowning commission

Officially titled *The New York State Temporary Commission to Prevent Childhood Drowning (NYS Drowning Commission)*, the NYS Drowning Commission was established in December 2021 by passage of NYS Assembly bill A.7734 and NYS Senate bill S.7129. The bills introduced by Assemblywoman Stacey Pheffer Amato and State Senator Timothy M. Kennedy were signed into law by Governor Kathy Hochul on 12/22/2021. Governor Hochul signed two extensions in June 2023 and in August 2024 to continue the NYS Drowning Commission through December 2025. The final report of the NYS Drowning Commission’s work was published in October 2025 [5]. Creation of the NYS Drowning Commission signaled the state’s commitment to address the issue of childhood drowning.

### Composition of the NYS drowning commission

The nine politically-appointed Commissioners were geographically diverse and included NYS Drowning Commission Chairwoman from the Mohawk Valley (Marrick McDonald, MS Ed, CAS), two commissioners from Western NY (Michael Switalski, MS; Kevin Clark, MS Ed), one from Central NY (Brian Tobin, MS), one from Northern NY (Gwen McNamara, MSOL), and four from NY City (Kaitlin Krause, BA; Casper Lassiter, MSW; Heidi Reiss; Kathryn Colglazier, BS). Diverse aquatic backgrounds included 307 combined years of aquatic experience gained in the for-profit, non-profit, public education, and higher education realms [5]. 

### NYS drowning commission activities

The NYS Drowning Commission’s activities were guided by five major goals as stipulated in the legislation (Table 1) [5]. 

**Table 1:** NYS Drowning Commission’s Goals

The NYS Drowning Commission (officially titled The New York State Temporary Commission to Prevent Childhood Drowning) was established by passage of NYS Assembly bill A.7734 and NYS Senate bill S.7129. The legislation contained the following five goals:
A. Determine an appropriate age and/or stage of childhood development when children can properly begin water safety and swimming instructions and develop statewide standards for such instruction
B. Investigate and evaluate the feasibility and effectiveness of programs which incentivize parents and guardians to enroll children in water safety and swimming instruction
C. Develop a comprehensive plan for public-private partnerships between the state and community centers, nonprofit organizations, recreational facilities, swimming instructors, and other relevant stakeholders and expand existing state resources such as parks and pools to provide access to free swimming instruction and determine the feasibility of such programs
D. Develop an implementation plan to ensure swimming safety programs are available in underserved communities
E. Develop a comprehensive plan for a public awareness campaign to ensure parents and guardians receive information on the importance of children receiving basic water safety and swimming instruction

 In fulfillment of these goals, the commissioners conducted more than 53 two-hour meetings and three full-day meetings. All meetings were conducted with a hybrid meeting option from an accessible public place with a virtual Webex option. Local and national water safety experts were invited to speak on numerous issues relevant to improving water safety and preventing drowning in NYS. In addition to the 21 formally invited presentations, numerous attendees from the NYS Department of Health, the NYS Office of Parks, Recreation and Historic Preservation, academics, and local aquatic and safety experts attended the biweekly sessions providing comments and answering commissioner questions. Public feedback was invited at the end of each of the biweekly meetings. Additional regional town halls were conducted to solicit feedback and garner community suggestions for water safety and drowning issues in their respective neighborhoods. Although the NYS Drowning Commission’s focus was to prevent childhood drowning, the sessions touched on issues related to all ages of drowning [5, 6]. Select key recommendations of the NYS Downing Commission’s 339-page report are shown in Table 2.

**Table 2:** Select key recommendations in the NYS Drowning Commission Report

Organizational Structure
• Create a central statewide office of water safety
• Create a statewide advisory network
• Develop a statewide database that captures data on fatal, nonfatal drowning and near-drowning incident characteristics
• Identify barriers to improving water safety
• Advance drowning prevention research
• Legislate lifeguards to be classified as first responders
• Develop a comprehensive plan for public-private partnerships
**Facilities/Equipment**
• Expand facilities and access to water safety training in resource-limited neighborhoods
• Build pools to increase access to water safety training
• Increase accessibility to safety equipment on beaches and natural bodies of water
• Protect existing public aquatic infrastructure by halting the closure and repurposing of pools
• Install clear signage in multiple languages near jetties, groins, and seawalls to explain the dangers of rip currents, backwash, and undertow
**Education/Standards**
• Establish age to begin water safety training for all children
• Develop a curriculum to integrate aquatic education for K-12 grades
• Expand water safety training in higher education
• Institute a mandatory college swim requirement
• Create aquatic safety minors and concentrations in higher education
• Expand number of lifeguards through more accessible and affordable training
• Identify and address needs of under-resourced, underserved, diverse, and special needs populations
• Strengthen boat safety and ice safety education to prevent drowning
• Develop water safety outreach, education, and promotion in multiple languages
• Create tangible incentives for individuals to participate in swim lessons
**Climate and Environmental Drowning Risks**
• Identify areas susceptible to drowning risks associated with climate change
• Enhance the early warning system for dams and levees
• Integrate advanced technology tools such as drones, sonar, AI surveillance, alarms, and remote-controlled buoys to enhance water safety and emergency response
• Develop flood-related drowning risk mitigation strategies including water alarms for basement apartments in flood prone areas

### Office of water safety

One of the key recommendations of the NYS Drowning Commission is the creation of an Office of Water Safety (Table 2). The proposed Office of Water Safety would function as a central clearinghouse to establish consistent standards, address existing and emerging water safety risks for residents and visitors, and provide a forum for transparency and collaboration across agencies, sectors, and industries. The creation of working groups to collaborate on water safety activities and mitigate issues as they arise would increase the ability of the state to meet the challenges posed by thousands of miles of coastline, growing climate resilience needs, intensifying weather events, and water hazards both on and offshore [5]. 

The dedicated Office of Water Safety would facilitate educational events and coordinate with partners to develop and deliver programs for diverse audiences, promote awareness, and water safety training. An aim of the Office of Water Safety would include promoting water proficiency, water competence and situational knowledge of water [5, 9]. The Office of Water Safety would support NYS as it adapts to evolving risks, including an improved approach to collecting, merging and housing a comprehensive database of fatal drownings, near drownings, and geographic features, such as housing and areas with characteristics associated with high drowning risk. A strategic framework is proposed that will improve drowning collaborations among stakeholders, such as the Statewide Agency Advisory Network, Aquatics Professional and Community Stakeholder Group, and the New York Water Safety Resource Platform [5]. 

### Statewide advisory network

To streamline the discussion, regulation and prioritization of water safety needs across the state, the NYS Drowning Commission recommends the creation of a statewide Advisory Network. The Advisory Network would consist of representatives from the Department of Health, Department of Environmental Conservation, Office of Parks, Recreation and Historic Preservation, educational institutions, community leaders, stakeholders, and other relevant parties. Working collaboratively, the Advisory Network would help develop and align a unified set of standards, regulations, and strategic priorities for aquatic and water-related issues related to drowning and water safety in NYS [5]. 

### Aquatic Professional & Community Stakeholders Group

The Office of Water Safety would facilitate formation of an Aquatic Professional and Community Stakeholders Group to serve as a forum for engaging water safety professionals and the aquatic community. The stakeholders’ group would work with the Office of Water Safety to convene periodic virtual meetings to share critical information, to discuss data issues, to collaborate on water safety initiatives, and to plan an annual statewide aquatic symposium. The annual symposium would function as a statewide networking event, a forum to share best practices and newly developing science, and as an avenue to discuss newly emerging issues in water safety.

### Integrating advanced technology to advance water safety

NYS faces growing risks from climate events including coastal surges, flash floods, and rip currents. The NYS Drowning Commission recommends that New York invest in and deploy advanced water surveillance and rescue technology across multiple at-risk environments, including beaches, rivers, flood zones as well as inland and urban areas at risk of flooding [5]. Specific technological tools recommended include water alarms in basement dwellings, remote-controlled buoys, AI powered surveillance, alarms for pools, crowdsourced flood mapping, portable air, sonar and underwater drones and enhancement of the early warning system for dams and levees [5]. 

### NY water safety resource platform

To centralize information and make it accessible to all New Yorkers, the NYS Drowning Commission recommends that the Office of Water Safety create an online resource platform to disseminate information on water safety education, swimming lessons, and other resources to promote water proficiency at both the community and organizational level. The platform would also host publicly available summary data on water-related injury and drowning to increase transparency and improve access to critical information for reduction of drowning and water-related injuries [5]. The Office of Water Safety would coordinate a wide array of educational initiatives for professionals and the public.

### Education of infants and children

The Drowning Commission adopted best practices from nationally recognized swim instruction organizations that water orientation classes and swimming lessons can begin as early as one-year of age with an accompanying parent, guardian, or caregiver [5, 10]. The American Academy of Pediatrics recommendations note that water orientation classes can begin as early as six months of age [10]. 

### Education of kindergarten to 12th grade

The NYS Drowning Commission recommends developing a curriculum to guide integration of aquatic education for students in Kindergarten to 12th (K-12) grade. This includes a recommendation to make waterless water safety education mandatory for K-12 school districts. Universal access to swimming instruction is recommended including an expansion of the NY City public schools Learn-to-Swim program, which currently requires second graders to receive a baseline level of skill-based education. It is recommended that the program be extended to all elementary grades and that a water safety elective be offered to secondary school students [5]. 

### Higher education

All NYS college students should be required to demonstrate an ability to swim either through passing a swim class or a swim test. The NYS Drowning Commission recommends several activities related to improving college/university aquatic instructor/professor standards. Individuals teaching aquatic education at institutions of higher education should have training, certification, or experience in teaching aquatic education. Aquatic administration training should be added into the degree/certificate requirements for majors in school administration and related degree/certificate programs. Physical education teacher majors should receive formal training on how to teach aquatics and have a baseline swimming ability equivalent to an American Red Cross Basic Swim Instructor, YMCA V6 Swim Instructor or the equivalent [5]. Colleges and universities should be encouraged to create aquatic minors/concentrations in teacher education programs (Table 2).

### Standards for swim instructors

The Drowning Commission recommends that all swim instructors have certification, experience, or standardized training and undergo background checks prior to providing swimming instruction. Instructors should be trained, certified or explerienced as an American Red Cross Basic Swim Instructor, YMCA V6 Swim Instructor or the equivalent [5]. Recommendations related to standardized aquatic knowledge and skills can be reviewed in the NYS Drowning Commission’s Report [5]. 

### Access to water safety education with incentivized participation

To address the low participation in swimming lessons in resource-limited communities, several incentives are recommended to facilitate parent and caregiver participation [5]. Especially important for resource-limited communities, provisions should be made as needed for free or affordable “swim essentials”: a swimsuit, cap, goggles, and, if desired, a shirt or culturally appropriate apparel to wear in the pool. Other incentives include free childcare for siblings during lessons and expanded class offerings during evenings and weekends to accommodate caregivers’ work schedules. Potential incentives included cash stipends, gift cards, metro cards, meals, or discounts on local services for families and individuals who enroll in and/or complete swimming instruction. Additional incentives included vouchers for additional water safety education /instruction and utilization of achievement markers such as patches, certificates, stickers and/or public recognition. The NYS Drowning Commission recommends working with local officials to develop transportation solutions as needed [5]. Collecting information on obstacles to participation in swimming lessons could facilitate development of a more effective incentive design structure.

### Data

NYS has several sources of epidemiologic drowning and near-drowning data that does not reside with a single agency or exist in a single accessible analytic data set. A coordinated, statewide approach is needed to develop a comprehensive system that can be used to inform decision making around water safety initiatives [5, 11]. Current data challenges include multi-sourced data, housed in separate databases frequently in separate organizations, and agencies using data collection forms that differ across agencies and organizations [5]. 

In addition to ongoing historical water safety issues, the NYS Drowning Commission recognizes that NYS, like many other states, has increased exposure to extreme weather and flooding [5]. There are data gaps in the information needed to address language barriers and housing prone to extreme weather flooding [5]. 

Current data on drowning or near drowning exists for hospitalizations and emergency department visits, emergency medical services, medical examiners, death certificates, school health, urgent care centers, Coast Guard, Beach Patrol, the Department of Environmental Conservation, the Office of Parks, Recreation and Historic Preservation and others. Health care visit data are protected by federal patient privacy laws that prevent the public release or posting of individual level health care data in a way that could be used to ascertain the identity of individuals. This makes it essential to retain the master drowning database containing these elements behind a secure firewall [5]. The NYS Drowning Commission recommends a single secure platform to house Health Insurance Portability Act (HIPAA) protected data. Once all data sources have been merged, cleaned and housed in a secure location, this data system could be used to create and release public summary reports of information to inform drowning interventions, program effectiveness, and drowning risk mitigation while adhering to federal laws that preserve patient privacy [5]. 

In addition to the individual level drownings or near drownings, data is needed on risk factors such as coastal features, pool facilities, lifeguard certifications, environmental conditions, flooding, housing and many other risk and protective factors. Currently, when they exist, these data are scattered across agencies making them or their format inaccessible for statewide or community-level policy, program and facilities planning. As an example of a seemingly unrelated housing characteristic, basement apartment dwelling continues to be a drowning risk factor in NYC. In NYC, 11 drowning deaths occurred in basement apartments during Hurricane Ida in 2021and two more similar deaths occurred in weather-related flash flooded basements in 2025. [7, 8]

### Recommended legislative initiatives

Data needs and recommendations could be accelerated using legislative initiatives. Currently, there is no accessible database of basement apartments prone to flooding and no requirement for water alarms in basement apartments susceptible to storm flooding. If installed in housing prone to flooding, these inexpensive devices would sound an alarm notifying occupants and neighbors in the event of water encroachment [5]. Other legislative recommendations include classifying lifeguards as first responders and mandatory posting of no swim signs near jetties or groins. Creation of mandatory waterless water safety education for K-12 public schools would benefit all but be particularly valuable for resource-limited neighborhoods without ready access to pools for swimming instruction [5]. Drowning safety initiatives successfully developed as a response to a NYS legislative mandate includes development and offering of a video to new parents during the mother’s hospitalization. This video increases the awareness of drowning risks for young parents many of whom have other children in the household [12]. 

### Underserved & under resourced areas

Drowning rates are disproportionately high in underserved and under-resourced communities. Additional recommendations included categories relating to scale up of accessible, multilingual aquatic education and water safety to very young children, elementary, middle school and high school students, adults, parents, guardians, and caregivers. Recommendations include identifying under-resourced communities, conducting outreach, education, and promotion in these communities, and providing financial assistance to make swimming lessons more accessible (Table 2) [5]. 

### Other recommendations

The NYS Drowning Commission report contains several recommendations related to safety equipment, boating safety, ice safety, pool safety, flood-related drowning risk mitigation strategies, and safety signage around jetties, groins, and seawalls [5]. 

### Progress on drowning prevention initiatives

A lack of accessible pool facilities is a major barrier to implementing the NYS Drowning Commission’s educational initiatives in under-resourced areas, under-served communities and education environments. Governor Hochul secured $150 million for drowning prevention capacity building and distributed it through a first round of 37 grants, with at least one drowning prevention grant awarded to every region of the state [13]. The Governor signed legislation requiring drowning prevention education be made available to the parents of every new birth. In fulfillment of this drowning prevention initiative, the NYS Department of Health produced a brief drowning prevention video that is offered to every new parent at NYS hospitals and birthing centers [12]. As part of the drowning prevention initiative, Governor Kathy Hochul unveiled *NY SWIMS: Statewide Investment in More Swimming* during her State of the State Address [13]. The primary aspect of the SWIMS initiative is the creation of more aquatic/swimming facility infrastructure.

### Summary

To address the continuing and newly evolving water-related safety risks, the NYS Drowning Commission recommends a unified, multi-pronged, equity-centered action plan that reaches across sectors and settings to build a culture of water safety. This plan provides the first comprehensive examination of NYS’s drowning risks across weather and climate events, geographic regions, environmental contexts, housing characteristics, disabled populations and resource-limited populations.

There are ongoing efforts to address the recommendations of the NYS Drowning Commission through legislative initiatives, stakeholder discussions, and community actions. Taken together, these measures provide a comprehensive blueprint for a safer NYS [14]. Governor Hochul aptly noted that “Access to swimming isn’t just about recreation – it’s about public health and climate resiliency.”

## Data Availability

The Commission’s final report and recordings of public meetings upon which the final report is based are available at: https:/www.ny.gov/programs/temporary-commission-prevent-childhood-drowning (Accessed 03/17/2026).

## Abbreviations

NYS: Drowning Commission The NYS Temporary Commission to Prevent Childhood
HIPAA: Health Insurance Portability and Accountability Act
K-12: Kindergarten through Twelfth Grade
NYS: New York State
NY SWIMS: New York Statewide Investment In More Swimming
WISQARS: Web-Based injury Statistics Query and Reporting System
